# Digitalisation and Employees’ Subjective Job Quality in the Second Half of Working Life in Germany

**DOI:** 10.1007/s11205-021-02854-w

**Published:** 2021-12-01

**Authors:** Lisa Katharina Kortmann, Julia Simonson, Claudia Vogel, Oliver Huxhold

**Affiliations:** 1 German Centre of Gerontology (DZA), Manfred-von-Richthofen-Straße 2, 12101 Berlin, Germany; 2 Neubrandenburg University of Applied Sciences, Brodaer Straße 2, 17033 Neubrandenburg, Germany

**Keywords:** Job satisfaction, Occupational stress, Subjective job quality, Digitalisation, German Ageing Survey

## Abstract

Since digitalisation alters occupational task profiles via automation processes, job quality is also likely to be affected. While existing literature mainly focusses on objective job quality, this study asks if and how digitalisation is associated with employees’ subjective job quality in the second half of working life in Germany. Analyses are based on the German Ageing Survey 2014. Our sample includes n = 1541 employees aged 40–65 years who are subject to social insurance contributions. Subjective job quality is operationalised with regards to job satisfaction and perceived occupational stress in general, and ten aspects of job quality in detail. Digitalisation is approximated by substitution potentials of occupations. We control the association for compositional effects in the workforce, as well as for the moderating effect of perceived job insecurity. The results indicate that digitalisation is predominantly beneficial but also unfavourable in a few other respects for employees’ subjective job quality. The higher the degree of digitalisation, the higher is the employee’s general job satisfaction on average; for general perceived occupational stress, we find no significant association. Regarding single aspects of subjective job quality, employees working in more digitalised occupations are found to report on average higher satisfaction with working hours and earnings, and lower levels of stress due to tight schedules. However, they also report higher levels of stress due to negative environmental factors.

**Supplementary Information:**

The online version contains supplementary material available at 10.1007/s11205-021-02854-w.

## Introduction

In recent decades, there has been a considerable increase in the adoption of new technologies in the labour market, which have automated occupational tasks. As a consequence, occupational profiles have changed and will continue to change profoundly. These changes may have had and will have substantial and far-reaching impacts on job quality. However, most empirical studies thus far have focussed either on single, objective aspects of job quality (e.g. wages; Fernández-Macías, 2012), on specific sectors (e.g. manufacturing; Körner et al., 2019) or specific technologies (e.g. computer- and internet-use; Kirchner, 2015). The technostress literature does investigate several aspects of subjective job quality, but its focus lies on the negative effects of technology usage (Bondanini et al., 2020). Currently, there is a lack of empirical studies that incorporate a comprehensive perspective.

This is surprising in many respects. First, politicians at the international and national level are paying increasing attention to job quality. In the OECD’s *Job Strategy*, launched in 2018, job quality is a central policy priority. At the national level, the German Federal Ministry of Labour and Social Affairs recently put the quality of jobs in the political spotlight by initiating the expert dialogue *Arbeiten 4.0 [work 4.0]*. Second, research has shown that low job quality is associated with outcomes such as lower productivity, poorer health and early retirement plans (Henseke, 2018; Royuela & Suriñach, 2013; Siegrist et al., 2006). Third, there is a broad consensus in the literature that job quality is a multidimensional concept (Cascales Mira, 2021; Muñoz de Bustillo et al., 2011), so any comprehensive analysis of job quality in light of digitalisation needs to consider multiple positive and negative job-related aspects. Fourth, most existing studies use only objective measures for job quality and do not capture individual preferences and subjective job assessments. Subjective measures of job quality can, however, provide valuable information about the employee’s well-being at work. Finally, for several labour-market-related decisions like job changes or the retirement transition, the employee’s subjective evaluation is of importance.

This study examines the potential impact of digitalisation on employees’ subjective job quality in the second half of working life in Germany. Specifically, we aim to answer the question: is working in more digitalised occupations associated with lower subjective job quality? To answer this question, we use representative data from the 2014 wave of the German Ageing Survey (DEAS, Deutscher Alterssurvey). The degree of digitalisation is indexed by the substitution potential—the ratio of potentially automatable tasks that are part of a particular occupation in relation to all tasks included in this occupational profile. We see the substitution potential as an approximation of the de facto degree of digitalisation in occupations. Subjective job quality is captured by job satisfaction and perceived occupational stress. We control for characteristics of the employee and the employee’s job that may be differentially affected by digitalisation to account for composition effects. Since job insecurity is one important aspect of job quality, and because it may be highly affected by the introduction of new technologies, we control for subjective job insecurity as well.

The main contributions of this work to the existing literature are: while an overwhelming number of studies on the implications of digitalisation for job quality focus on objective, one-dimensional, or one-sided measures, this study provides a subjective, multidimensional perspective on job quality, considering positive and negative aspects. Furthermore, we use a comprehensive measure for digitalisation that contains all new technologies, not only computer use or robot density. Finally, we focus on employees in the second half of working life. Thus, we investigate a group that might be particularly vulnerable to digitalisation as their skill sets may have a higher likelihood of becoming fully or partly outdated due to digitalisation. Younger employees who recently entered the labour market are typically better educated, more digitally savvy and may possess better skills to deal with new technologies. Most importantly, in recent years there have been a number of policy measures aimed at prolonging working life. These policies aim to cope with challenges to the labour market and pension system due to the ongoing process of demographic change. Poor job quality could counteract these efforts.

In comparison with the OECD average (9%), the share of employees working in occupations with a high automation potential^1^ was among the highest in Germany in 2012 (12%) (Arntz et al., 2016). Furthermore, in 2014 every second employee in Germany (52%) used a computer with internet access at work—which was above the European average (46%) (OECD, 2021).

## Previous Research

### Digitalisation and the Labour Market

In this study, we use the term “digitalisation” to refer to the spread of digital devices, applications and machines that started in the second half of the twentieth century. One theoretical perspective on digitalisation’s impact on employment is the task-based approach (TBA), coined by Autor et al. (2003). It assumes that routine tasks can be automated by digital technologies. Non-routine tasks, in contrast, cannot be fully translated into algorithms at present. In recent literature, the TBA has been used to determine the degree of “automatability” in occupations. While some studies imply that digitalisation will lead to full automation of jobs and thus to the replacement of workers by digital technologies (Frey & Osborne, 2017), others emphasized that digitalisation will mostly lead to changes in occupational task profiles (Dengler & Matthes, 2018). In line with their emphasis, we prefer the use of the comprehensive and neutral term of “digitalisation”.

### Digitalisation and Subjective Job Quality

Regarding the impact of digitalisation on job quality, two opposing positions have been taken in the field. Some studies have noted the beneficial aspects of technologies. For example, new technologies are seen as key drivers for increasing flexibilisation of the organisation of work as they facilitate the implementation of telework, home office or flexible working time arrangements. Flexible working time arrangements have been found to have positive effects on employees’ work-life balance (Hill et al., 2001). A good work-life balance is in turn positively associated with job satisfaction and negatively associated with mental health issues (Haar et al., 2014). Additionally, digitalisation may reduce the burden of physical labour. Certain technologies, typically industrial robots, are built to execute physically demanding tasks, like lifting heavy loads, or replacing humans in harsh environmental conditions. This could alleviate physical stress and improve occupational safety, thus preventing work-related illnesses (Theurel & Desbrosses, 2019). Other technologies, typically computers, execute cognitive demanding tasks, like complex calculations.

However, the empirical evidence concerning the impact of digitalisation on job quality is not uniformly positive. The field of technostress research focusses in particular on the negative relationship between employees’ psychological well-being and the use of new technologies (Bondanini et al., 2020). Such studies have identified different stressors, like inadequate skills to deal with new technologies, or the fear that one’s job will be substituted (Tarafdar et al., 2011). The rise of new technologies has led to an intensification and acceleration of work. Faster communication, information, production and distribution can lead to higher stress levels in employees (Green, 2004). In line with this, Germany has experienced an increase in high speed work and working to tight deadlines in recent years, coinciding with a slight decrease in job satisfaction over the same time period (Hoonakker, 2014). Furthermore, digitalisation is related to the blurring of boundaries between working and private life. Felstead and Henseke (2017) found remote working associated with an intensification and greater difficulties switching off from work. Employees’ physical well-being can be negatively affected as well. Constant computer use at work has been found to be related to physical strain (Andries et al., 2002). The use of new technologies can lead employees to work in unhealthy physical postures, as occurs when spending extended periods of time sitting or working at a screen. Long hours of computer work have, for example, been found to be related to wrist and arm problems (Gerr et al., 2002, 2006; Jensen, 2003).

#### Subjective Job Quality

The concept of job quality refers to aspects that contribute to fulfilling employees’ work-related needs or ensuring their well-being (Brown et al., 2007; Green, 2006; Muñoz de Bustillo et al., 2011). There are objective and subjective perspectives on job quality. The objective perspective focusses on a bundle of observable job characteristics, like earnings or working time arrangements. In contrast, a subjective job quality perspective focuses on employees’ assessments of their job. A subjective perspective considers how characteristics of the occupation are evaluated and prioritised by the employee in accordance with their individual needs, preferences and experiences. One simple way to depict job quality is to use a global measure of job satisfaction (Clark, 2011; Green, 2006). However, this approach has been criticised for oversimplifying the multidimensionality of the concept (Muñoz de Bustillo et al., 2011).

Therefore, in this study we take job satisfaction and perceived occupational stress as dimensions of subjective job quality. Job satisfaction and stress are commonly used dimensions to depict subjective job quality (Handel, 2005; Olsen et al., 2010; Soh et al., 2016; Warr, 1999). The association between occupational stress and job satisfaction is not as straightforward as it might seem; while higher levels of stress are generally associated with lower levels of satisfaction, some aspects of stress could be associated with higher levels of job satisfaction. In particular, perceived stress does sometimes acts as a motivator, hence occupations can be experienced as both stressful and satisfying at the same time. Some studies distinguish between challenge and hindrance stressors, which show positive or negative associations with work related aspects (Podsakoff et al., 2007). High workloads, for example, are found to be associated with higher levels of satisfaction with the variety of work tasks and activities or with the salary (Burke, 1976). Therewith, a high workload could be classified as challenge stressor.

Job satisfaction describes workers’ overall contentment with different characteristics of their job. Perceived occupational stress describes workers’ physical or psychological reaction to a mismatch between their expectations and their actual capacity to meet different work-related demands (Kahn et al., 1964). Job satisfaction and perceived occupational stress can be conceptualised either as global or multidimensional measures. Global measures are either based on single-item questions—e.g. ones that asks employees to assess their satisfaction with their current job in general, or by index measures, summing up employees’ assessments towards different aspects of their job. Single-item global measures have the advantage that they rely on the employee’s preferences and relevance systems, so the selection and weighting of different job aspects is up to the employees themselves. Index global measures, based on employees’ assessments towards a pre-selected list of different job-related aspects, raise the problem of how to weight different aspects.

Equal weights are usually applied to all pre-selected aspects of a job in order to build an index. A multidimensional measure of job satisfaction describes employees’ satisfaction with different aspects of their job: e.g. satisfaction with earnings, the kind of work, or opportunities for promotion or career development. A multidimensional measure of perceived occupational stress is comprised of different stressors related to a job, which can be physical or psychosocial in nature. Physical stressors may include monotonous work, high paced work, work in noisy environments or work under extreme climatic conditions. Psychosocial stressors may include bad relationships with colleagues or superiors, time pressure or ambiguous working instructions.

In contrast to global measures, a multidimensional perspective allows to investigate different facets of a concept that are differentially affected by a given phenomenon. Therefore, in this study, we use multidimensional measures for job satisfaction as well as for perceived occupational stress and consider each facet separately in addition to using global measures for job satisfaction and occupational stress.

#### Subjective Job Quality and Digitalisation

The association between subjective job quality and digitalisation is to date an understudied topic, especially when it comes to studies using representative data and comprehensive measures for both. For example, we only found few studies that used broad multidimensional measures of job quality and identified a link between job quality and digitalisation (Handel, 2005; Olsen et al., 2010). However, both studies only implicitly linked subjective job quality to digitalisation and did not incorporate a measure of digitalisation in the analyses. While Handel (2005) focussed on trends in perceived job quality in the US over time, Olsen et al. (2010) investigated differences between and within countries at different time points. Overall, both studies mostly demonstrated stability in perceived job quality measured by job satisfaction over time. Moreover, looking at single aspects of job quality, both studies found few significant changes. Specifically, findings pointed to improvement over time for some aspects of job quality and deterioration for others.

In contrast to these approaches, Gallie (2003) included “working with new technologies” as a direct measure of digitalisation. He found that working with new technologies was associated with higher job quality in 1996 in Europe. Unfortunately, it remains unclear what “working with new technologies” actually measured, since the study lacked an extensive description of the item. However, it can be assumed that the understanding of “new technologies” was up to the survey respondents themselves, and as a consequence the actual impact of working with new technologies was not uniformly depicted. In addition, this study considered only four aspects of subjective job quality: the quality of working tasks, involvement in decision making, career opportunities and job security. Important aspects such as stress in the workplace, earnings, or relationships with colleagues or with the boss were not considered.

Dengler and Tisch (2020) used a comprehensive measure of digitalisation but a rather restricted measure for subjective job quality. The authors investigated the association between potential digitalisation and overall physical and psychosocial exposure at work in Germany. The measures for subjective job quality only focussed on perceptions of stress and did not cover other aspects of satisfaction with a job. Their findings indicated that new technologies may relieve men of physically demanding work. Since institutional frameworks and labour market preconditions are relevant factors impacting on subjective job quality (Gallie, 2007), a distinct investigation of Germany using an extensive measure of subjective job quality is needed.

#### Subjective Job Quality, Digitalisation and a Composition Effect

As described above, occupations are differentially affected by digitalisation, and employees with certain sociodemographic and job-related characteristics vary systematically across different occupations. For example, substitution potentials are found to be highest in the manufacturing sector, occupations concerned with production technology or occupations in business management and organisation. Occupations in cleaning services, safety and security or service occupations in the social or cultural sector show the lowest substitution potentials (Dengler & Matthes, 2018). Consequentially, it is likely that differences in subjective job quality are not solely due to differences in the degree of digitalisation at work but are at least partly due to employees’ sociodemographic and job-related characteristics. Thus, in line with the studies on subjective job quality and digitalisation mentioned above, we consider the following variables as important control variables: age, gender, residency in East or West Germany, educational level, sector of employment, company size, health status, hourly wage and working hours. The theoretical rationale for including these characteristics is detailed below.

It is well known that men and women are distributed differently among occupations in the German labour market (Busch, 2013); Male-dominated occupations have been found to show higher average potential degrees of digitalisation than female-dominated occupations (Piasna & Drahokoupil, 2017). Regarding job satisfaction, women are found to show on average higher satisfaction than men (Perugini & Vladisavljević, 2019). However, studies on the association between occupational stress and gender show ambiguous findings (Gyllensten & Palmer, 2005). Regarding education and wages, it has been demonstrated that lower degrees of digitalisation correlate with higher educational levels and higher individual wages of employees (Dengler & Matthes, 2019). Tasks that are not automatable are often more complex and require higher skills from the employee. Higher educational levels and higher wages may reflect these needs. At the same time, highly educated people usually work in jobs that are objectively characterised by good job quality: they are usually employed in higher paid jobs, have more autonomy at work, and their work is less physically demanding. Employees’ age can be assumed to be related with the occupations’ potential degree of digitalisation and subjective job quality. Studies have found that older employees are expected to be less willing and capable to learn new things (Hauk et al., 2018; Warr & Birdi, 1998), and that computer and internet use at work is lower among older individuals (Huxhold et al., 2020). Thus, older employees may be less exposed to new digital devices and applications at the workplace. Despite this, with increasing age employees are found to show lower levels of occupational strain due to digital technologies (Hauk et al., 2019; Ragu-Nathan et al., 2008), and higher levels of job satisfaction (Ng & Feldman, 2010). Therefore, we expect job satisfaction to increase and occupational stress to decrease with age in our analyses. The sector of employment is related to the potential degree of digitalisation. The industry sector is characterised by repetitive working processes in protected work environments (e.g. factories) that provide favourable circumstances for new technologies. Furthermore, different sectors may be related to different average levels of job satisfaction or occupational stress, since working conditions may differ considerably. Industrial jobs can be often characterised by a noisy working environment, assembly line work and monotonous working tasks. In contrast, jobs in the public sector often have more favourable working conditions, like good working time regulations or opportunities for further training. Despite German reunification in 1990, East-West differences in job satisfaction and occupational stress seem to persist (Datenreport 2018, 2018). Furthermore, company size can be expected to be related to digitalisation and job quality. Bigger companies may have greater financial resources to implement new technologies in the company. Regarding job quality, smaller companies have been found to provide less favourable working conditions compared to larger companies, for example, lower wages or fewer opportunities for skill enhancement (Wagner et al., 2002). However, a more familiar atmosphere and shorter communication channels in small companies may compensate for such objectively less favourable working conditions and subjective assessments of working conditions may differ in that respect. The time spent at work may be determined by the employment contract (e.g. part-time) or by individual engagement (e.g. overtime). While there are only little differences between part- and full-time employees regarding (aspects of) job satisfaction (Thorsteinson, 2003) long working hours correlate with several negative health outcomes (Bannai & Tamakoshi, 2014). Lastly, we consider self-rated health (SRH) as an important control variable in our analyses, as it is likely to be related to digitalisation as well as subjective job quality. Employees with poor health can be assumed to self-select into jobs with higher degrees of digitalisation, since new technologies may compensate for their physical deficiencies. In addition, SRH is an important predictor of subjective well-being (Smith et al., 2010) and job satisfaction and occupational stress are closely related to a person’s overall well-being.

#### Job Insecurity, Subjective Job Quality and Digitalisation

In research on job quality job insecurity is named as one crucial determinant with job insecurity showing a negative association with job satisfaction and a positive with occupational stress (Ganster & Rosen, 2013; Hur, 2019; Sverke et al., 2002). Some longitudinal studies demonstrate that workers in occupations that are highly impacted by digitalisation have a higher probability of becoming unemployed (Biagi et al., 2018; Cortes, 2016). Hence, the degree of digitalisation of occupations can be expected to affect subjective job insecurity. In fact, we found a low but significant positive correlation between the degree of digitalisation with subjective job insecurity (Spearman’s rho = 0.115). Since we are interested in the impact of new technologies on employees’ assessments of their working conditions in their current occupation, regardless of any risk of job loss, we control for subjective job insecurity.

### The Current Study

This study aims to answer the following overarching question: how is the digitalisation in occupations associated with the subjective job quality of employees aged 40 and older in Germany? Subjective job quality is measured by job satisfaction and perceived occupational stress. We focus on employees in the second half of their working life for different reasons. First, it can be assumed that due to their long past training phase, a higher share of their skills and knowledge might have become outdated or even obsolete in times of technological change. For older employees, there is less time for training to pay off before they retire. Consequently, broad changes in the occupational task profiles may considerably burden employees in the second half of their working life. Second, employees in the second half of their working life usually have some years of experience in the labour market and may be settled in a job that suits their ideas and needs.

#### Hypotheses—Digitalisation and Job Quality

We expect higher degrees of digitalisation in occupations associated with lower levels of the job satisfaction *index* (H1.1), as well as with job satisfaction *as a whole* (H1.2). Furthermore, we expect higher degrees of digitalisation associated with higher levels of the occupational stress *index* (H2). This is because, if not controlled for compositional effects, we assume more digitalised occupations to be characterised by aspects typically associated with lower levels of job satisfaction or higher levels of occupational stress. Since the implementation of new, digital technologies in occupations is ever-progressing, employees working in more digitalised occupations may on average still assess their job quality as lower than employees in occupations that are less digitalised.

With respect to specific aspects of job quality, we expect more digitised occupations to be associated with lower levels of satisfaction with *opportunities for further training* provided by the employer (H1.a). On one hand, employees facing new technologies may see a greater need for further training. On the other hand, employers may shy away from investing in employees in the second half of life. In contrast to younger employees, older employees have shorter time horizons on the labour market and are not digital natives. Further, we hypothesise that employees working in more digitised occupations report higher levels of stress due *to strenuous, physical work* (H2.a) and *negative environmental factors* (H2.b). This is because we expect to find higher digitalisation degrees in those occupations with more arduous or harsh working conditions. Therefore, potentially positive effects of new technologies might not be revealed if compositional effects are not controlled for.

#### Hypotheses—Digitalisation and Job Quality While Controlling for Compositional Effects

Controlling for compositional effects of the workforce and based on the literature and considerations mentioned above (Sects. 2.2.2 and 2.2.3), we expect that higher degrees of digitalisation of occupations are associated with higher levels of the job satisfaction *index* (H3.1) and job satisfaction *as a whole* (H3.2), as well as with lower levels of the occupational stress *index* (H4). The reasoning behind these assumptions is that compositional effects of the workforce may overshadow a positive effect of new technologies on global measures of job satisfaction and occupational stress.

In addition, we hypothesise that, controlling for compositional effects, employees in more digitalised occupations are less satisfied with opportunities for *further training provided by the employer* (H3.a). The rational corresponds to that of H1.a. Further, digitalisation has been shown to lead to higher flexibilisation, such as flexible working time arrangements, and to be related to higher autonomy at work. Therefore we expect to find higher levels of satisfaction with *working hours* (H3.b) and higher levels of satisfaction with the *working atmosphere* (H3.c) for employees working in more digitalised occupations. The satisfaction with the *kind of work* is expected to be higher in more digitalised occupations (H3.d), since new technologies may substitute for or alleviate some stressful working tasks. We expect to find employees reporting lower levels of stress due to *strenuous physical tasks (H4.a)* and lower stress due to *negative environmental* factors (H4.b) the higher the degree of digitalisation. This is because new technologies may alleviate physically demanding tasks or harsh working conditions. Digitalisation has been shown to lead to an acceleration of working processes. Therefore, after controlling for compositional effects, we expect employees working in more digitalised occupations to experience more stress due to *tight schedules* (H4.c). Furthermore, we expect higher levels of stress due to *new responsibilities* (H4.d) to be linked with higher degrees of digitalisation, since digitalisation processes may lead to large and constantly new changes in working tasks requirements.

#### Hypotheses—Digitalisation and Job Quality while Controlling for Compositional Effects and Job Insecurity

As digitalisation can lead to job losses due to substitution processes, subjective job insecurity may be higher among employees working in occupations with higher degrees of digitalisation. If employees see their job at risk, negative attitudes towards their job may be higher compared to employees who feel their job is secure. Therefore, we expect employees working in more digitalised occupations who do not feel threatened by substitution to show higher levels of the job satisfaction *index* (H5.1), higher levels with job satisfaction *as a whole* (H5.2) and lower levels of the occupational stress *index* (H6). Regarding single aspects of job satisfaction and perceived occupational stress, we expect to find associations similar to those formulated in the hypotheses for the compositional model (H5.a–H5.d & H6.a–H6.d). Furthermore, we hypothesise that higher levels of *subjective job insecurity* are associated with lower levels of the job satisfaction *index (H7.1),* job satisfaction *as a whole* (H7.2), and all aspects of job satisfaction (H7.1–H7.f). Lastly, we expect that the higher the *job insecurity*, the higher the levels of the occupational stress *index* (H8) and all aspects of occupational stress (H8.a–H8.d) (for an overview of all hypotheses see supplemental material, Table 14).

## Method

### Sample

To answer the research question, we used data from the baseline sample of the 2014 wave of the German Ageing Survey (DEAS) (Klaus et al., 2017). The DEAS 2014 is a cross-sectional and longitudinal survey representative for the resident population of private households aged 40 and above in Germany. The baseline sample is based on registration office data, is stratified by age, gender and place of residence, and comprises 6002 respondents in 2014. The DEAS is based on a standardized, computer-assisted personal interview that has been conducted by professional interviewers in the respondents’ homes. It contains detailed information on the living and working situations of people in the second half of life as well as assessments of different aspects like job satisfaction or occupational stress. Furthermore, we used data on the digitalisation of occupations, namely substitution potential, calculated by the IAB.

The analyses investigated employees between the ages of 40 and 65 that are subject to social insurance contributions. Individuals that were not employed full- or part-time and those aged 66 and above were excluded from the sample. Furthermore, individuals with imprecise occupational information (that is ISCO codes less precise than unit group level) were excluded, as detailed information on the occupation was essential for the analyses. The sample was comprised of *N* = 1541 employees (49% female; *M*_age_ = 51.8; *SD*_age_ = 6.15) and showed a slight overrepresentation of highly educated employees, a bias documented also for the general DEAS 2014 sample (Klaus et al., 2017) (see supplemental material, Table 1).

### Measures

#### Dependent Variables

Subjective job quality was captured by two dimensions; job satisfaction and perceived occupational stress. Besides index measures of both dimensions, both measures were broken down into their sub-dimensions to get a more detailed picture.

*Job satisfaction:* We used a job satisfaction index built from six items on employees’ assessments towards different aspects of their job, stated on a 5-point Likert-scale (McDonald’s omega: 0.75). The different aspects of job satisfaction concerned earnings, the job itself, working hours, opportunities for career development or promotion, opportunities for further training offered by the company and the working atmosphere. In addition satisfaction with employees’ work as a whole was considered. The answer scale ranged from “very satisfied” to “very unsatisfied” and was inverted, such higher values indicate more satisfaction. On average, employees reported being “rather satisfied” with their job; the mean of the job satisfaction index was 3.83, while the mean of job satisfaction as a whole was slightly higher (4.16).

*Occupational stress:* In DEAS 2014 four aspects of occupational stress were assessed: Employees perceived stress due to physical activities, negative environmental factors, heavy workloads or tight deadlines, and new job responsibilities. The 5-point response scale ranged from “very stressed” to “not at all stressed” and was inverted. The single aspects were additionally aggregated to an index (McDonald’s omega: 0.61). On average, employees reported being slightly stressed (mean index: 2.85).

#### Independent Variables

*Digitalisation:* To depict the occupations’ degree of digitalisation, we used substitution potential determined by the Federal Institute of Employment Research (IAB, Institut für Arbeitsmarkt- und Berufsforschung) (Dengler & Matthes, 2018). This was calculated by dividing the share of occupational core tasks that were potentially automatable by the share of all core tasks of the same occupation. If an occupational task was considered as automatable or not relied on independent judgements from three experts. In our study, we interpreted substitution potential as a measure of the degree of digitalisation thrust in occupations. We assume that occupations with higher substitution potential have already undergone processes of digitalisation to a larger degree than occupations with lower substitution potential. We were able to assign a valid substitution potential value to 95 percent of the sample. For the remaining five percent a meaningful value was imputed. Occupations with high degrees of digitalisation are, for example, clerical workers or machine operators in the production; Teachers or bus drivers are occupations assigned to low degrees of digitalisation.

*Job insecurity:* Subjective job insecurity is measured by asking about the respondents’ appraisal of the likelihood of losing their job in the near future. The 4-point answer scale ranged from “very likely” to “very unlikely”. Job insecurity was slightly positively correlated with occupations’ degree of digitalisation (0.1 Spearman’s rho). Overall, employees assessed the likelihood of job loss in the near future as rather low (mean: 1.65).

#### Control Variables

We controlled for the stratification variables of age, gender and residency underlying the DEAS sample. Age was measured in years and added as a continuous variable to the analysis. Gender and residency were added as dummy variables, with “1” for “male” “living in West Germany”^2^ respectively. We also controlled for job characteristics to address a potential composition effect—here, we looked at educational level, sector, firm size, SRH, hourly wage and working hours. We controlled for the educational level using a three-level classification based on the International Standard Classification of Education (ISCED) to distinguish between a low, middle and high educational level (OECD et al., 2015). A low educational level was used as the base category. Regarding information on the sector an occupation is located in, DEAS data allows differentiation between five major sectors: agriculture and forestry, industry, handicraft, commercial and service and public service. We added the sector as a categorical variable to our analysis (agriculture and forestry, industry, handicraft, commercial and services, public services) with “agriculture and forestry” as base category. Company size was measured by the number of employees and ranges from “1” for “less than five employees” to “5” for “more than 2000 employees”. We used SRH measured by the single question “How do you rate your current state of health?” to control for the employees’ health. The 5-point answer scale ranges from “very good” to “very bad” and was inverted for a more intuitive interpretation. Hourly wages were added as a continuous variable to our analyses. They were determined by dividing the monthly net income by working hours. This gave us a measure for the monetary valuation of work independent of differences in working hours. To control for differences in working hours specifically, we added information on employees’ weekly working hours, including overtime to our analyses. There was no indication of high multicollinearity among the considered variables (Cohen, 1988).^3^

### Analyses

To analyse the potential impact of digitalisation on job quality, we estimated three multiple linear regression models for each dependent variable and reported unstandardised partial regression coefficients. In the first model—the pure model—we estimated the relationship between job satisfaction or, perceived occupational stress and digitalisation, while controlling for age, gender and residency to account for the disproportional sampling in DEAS. In the second model—the compositional model—we added information on education, sector, company size, SRH, wage and working hours to control for compositional effects within the workforce. In the third model—the job insecurity model—we also controlled for subjective job insecurity.

To make full use of the data, multiple imputation was used to impute missing values on all variables considered in our analyses (Rubin, 1987). Specifically, multiple imputation with chained equations (MICE) and M = 50 imputation models were applied. We used chained equations, as the missing pattern in the data is multivariate and not monotonous. Additionally, some of the variables containing missing values were not continuous. We applied several tests suggested in the literature to check the quality of the imputation (Nguyen et al., 2017). Some robustness tests performed substantiate the stability of our findings (see supplemental material, Tables 10–13). We present the results of our analyses in the following section.^4^ An association is considered to be statistically significant if the coefficient’s *p*-value value falls below 0.05.

## Results

### Digitalisation and Job Quality

In the pure model, we found no significant associations between higher levels of digitalisation in occupations and the *index* measure of job satisfaction (b = 0.009; *p* = 0.889) or employees’ satisfaction with their *work as a whole* (b = 0.092; *p* = 0.233). Similarly, no significant association of digitalisation with the *index* measure of perceived occupational stress was found (b = 0.106; *p* = 0.186). Therefore, hypotheses H1.1, H1.2 and H2 have to be rejected.

Regarding single aspects of job satisfaction, higher degrees of digitalisation were associated with higher satisfaction with *working hours* (b = 0.429; *p* = 0.000); yet, higher digitalisation was linked to lower levels of satisfaction with *opportunities for further training* (b = -0.362; *p* = 0.004). Therefore, hypothesis H1.a was confirmed. In line with our hypotheses H2.a and H2.b, we found higher degrees of digitalisation related to more stress due to *physical activities* (b = 0.381; *p* = 0.002) and due to *negative environmental factors* (b = 0.810; *p* = 0.000). Lastly, we found digitalisation negatively associated with stress due to tight schedules (see Table 1, p. 18).

### Compositional Effects

In the composition model, we found positive associations of digitalisation with both global measures of job satisfaction (*index*: b = 0.172; *p* = 0.019; job satisfaction *as a whole*: b = 0.181; *p* = 0.035), but no association with the index measure of occupational stress (b = -0.034; *p* = 0.693). Therefore, hypotheses H3.1 and H3.2 were confirmed, but hypothesis H4 not.

Regarding single aspects of job quality, we found support for hypothesis H3.b. Working in more digitalised occupations correlates with higher satisfaction with *working hours* (b = 0.465; *p* = 0.000). Further, employees working in occupations that are digitalised to higher degrees are more satisfied with their *earnings* (b = 0.253; *p* = 0.020) as well as with *opportunities for career development* (b = 0.249; *p* = 0.045). Hypotheses H3.a, H3.c and H3.d were not confirmed (*further training*: b = -0.109; *p* = 0.448; *working atmosphere*: b = 0.103; *p* = 0.318; *kind of work*: b = 0.069; *p* = 0.462). Regarding aspects of perceived occupational stress in the compositional model, higher levels of digitalisation were found to be associated with more stress due to *negative environmental factors* (b = 0.345; *p* = 0.011), but less stress due to *tight schedules* (b = -0.518; *p* = 0.000). Thus, the results were contrary to our expectations formulated in hypotheses H4.b and H4.c. No significant association was found for digitalisation with occupational stress due to *strenuous physical tasks* (b = -0.055; *p* = 0.667) and due to new responsibilities (b = 0.093; *p* = 0.465). Consequently, hypotheses H4.a and H4.d were also rejected.

While the educational level of employees does not seem to have a great impact on (aspects of) job satisfaction, it appears as important control variable regarding (aspects of) occupational stress. High educated employees report on average lower stress due to physical or environmental factors, but higher stress due to tight schedules and new responsibilities at work. Furthermore, job satisfaction and stress are found to be impacted significantly by the size of the company, employees’ health status and wages. Bigger companies seem to be associated with lower job satisfaction and higher stress among employees. Contrary to that, the employees’ health status and wages show positive associations with (most aspects of) job satisfaction and occupational stress (see Table 1, p.18).

### Job Insecurity

As in the compositional model, and in line with hypotheses H5.1 and H5.2 for the job insecurity model, higher degrees of digitalisation were associated with higher levels of job satisfaction, measured by global measures (*index:* b = 0.188; *p* = 0.009; *as a whole*: b = 0.198; *p* = 0.018). Likewise, hypothesis H6 was rejected, as no significant association was found for the occupational stress *index* (b = -0.040; *p* = 0.639).

Controlling for perceived job insecurity and compositional effects, we found that higher degrees of digitalisation associated with more satisfaction with *working hours* (b = 0.473; *p* = 0.000) and less stress due to *tight schedules* (b = -0.522; *p* = 0.000). Hence, hypothesis H5.b was confirmed but H6.c had to be rejected. Contrary to hypothesis H6.b, we found a significant relationship between working in more digitalised occupations and higher levels of stress due to *negative environmental factors* at work (b = 0.336; *p* = 0.014). We also found positive associations between higher degrees of digitalisation of occupations with employees’ satisfaction with *earnings* (b = 0.266; *p* = 0.014) and with *opportunities for career development* (b = 0.274; *p* = 0.025). Hypotheses H5.a, H5.c, H5.d and hypotheses H6.a and H6.d were not confirmed, as no significant associations were found (*further training*: b = -0.081; *p* = 0.568; *working atmosphere*: b = 0.116; *p* = 0.260; *kind of work*: b = 0.081; *p* = 0.384; *physical activities*: b = -0.060; *p* = 0.639; new responsibilities: b = 0.085; *p* = 0.504).

Furthermore, our results indicate that employees feeling more at risk of losing their jobs reported lower levels of job satisfaction, measured by global measures (*index:* b = -0.165; *p* = 0.000; *as a whole*: b = -0.180; *p* = 0.000) or regarding single aspects of job satisfaction (*further training*: b = -0.285; *p* = 0.000; *working hours*: b = -0.073; *p* = 0.036; *working atmosphere:* b = -0.128; *p* = 0.000; *kind of work*: b = -0.124; *p* = 0.000; *earnings*: b = -0.130; *p* = 0.000, career opportunities: b = -0.248; *p* = 0.000). Therefore, hypotheses H7.1–H7.f were confirmed A positive association with the f perceived stress *index* (b = 0.065; *p* = 0.021) as well as with stress due to *environmental factors* (b = 0.091; *p* = 0.043) was found. An association significant on 90 percent confidence level was found for stress due to *new responsibilities* and job insecurity (b = 0.081; *p* = 0.054). Therefore, hypotheses H8, H8.b and H8.d were confirmed, but hypotheses H8.a and H8.c were rejected (*physical activities*: b = 0.051; *p* = 0.231; *tight schedules*: b = 0.036; *p* = 0.347) (see Table 1, p.18).

## Discussion

In sum, higher degrees of digitalisation in occupations may lead to both improvements and encumbrances for employees in the second half of working life in Germany, such as more occupational stress due to negative environmental factors. However, overall job satisfaction is higher for employees working in more digitalised occupations. These findings are in line with related studies on job quality in times of digitalisation (Handel, 2005; Hoonakker, 2014; Olsen et al., 2010).

### Digitalisation and Job Quality

Overall, employees in more digitalised occupations differ in a few aspects of job satisfaction from employees in less digitised jobs. They are more satisfied with their working hours. This may result from greater flexibility in working time arrangements and opportunities to work from home facilitated by new technologies. In line with this, digitalisation is associated with lower levels of stress due to tight schedules, deadlines or nervous tensions. This result seems surprising, since in the literature digitalisation has been associated with higher stress levels due to an intensification and acceleration of work (Green, 2004; Green & McIntosh, 2001). However, one explanation—in line with the findings of Shultz et al. (2010)—could be that those employees enjoy more flexibility to organise their work. Thus they are provided with more room for manoeuvre to deal with tight schedules and deadlines and are consequently able to cope with the acceleration of work. Furthermore, employees working in more digitalised occupations report lower satisfaction with opportunities to take part in employer-provided further training. However, this association was found in the pure model only. This may be because more digitalised jobs involve less training. Contrary to our expectations, perceived stress due to strenuous physical activities and negative environmental factors was on average higher among employees working in more digitalised occupations. It can be assumed that more digitalised occupations are not only those with inherently worse opportunities for further training but also those with worse working environments or more physically demanding work.

### Compositional Effects

Our findings show that controlling for compositional effects is important when investigating subjective job quality. More digitalised occupations were characterised by lower subjective job quality or at least their employees were more likely to have characteristics usually associated with low job quality. Because under control of compositional effects, we find the job satisfaction index and satisfaction with work as a whole positively associated with more digitalized occupations. The negative association with employees’ satisfaction with further training is not significant after controlling for compositional effects. Corresponding to the findings of the pure model, satisfaction with working hours is higher and stress due to tight schedules is lower when working in more digitalised occupations. And the higher the degree of digitalisation in occupations the higher the satisfaction with career prospects or promotion. Since digitalisation processes in occupations rely on transformation processes, old structures may be revised and new opportunities for career development may open up. Another explanation could be that digitalisation leads to a higher demand for skilled workers who are able to create, serve or maintain new technologies or evaluate and analyse data collected by new technologies (Goldin & Katz, 2008; Goos & Manning, 2007). Moreover, the compositional model also shows employees working in more digitalised occupations reporting higher stress due to negative environmental factors. The assumed inherent higher physical strain and worse working environments in more digitalised occupations seems to partly explain the reported higher stress due to onerous physical and environmental factors. A further explanation could be that new technologies bring along some negative work environments such as noise, bright screens or flashing light signals. Industrial robots working at high speed may create loud noise and employees working frequently with video screens may perceive stress due to overly dim or bright light. These results are consistent with findings from other studies that found an increase in hard work and stress due to physical work or higher exhaustion over time (Handel, 2005; Olsen et al., 2010). Lastly, even if wages and other factors are assumed to be equal between employees, those working in more digitalised occupations are more satisfied with their earnings. Employees in more digitalised occupations may experience their work as less burdensome overall and respectively more rewarding in terms of non-monetary aspects. Therefore, earnings may be less of a compensation for onerous experiences at work if satisfaction with other work-related aspects is high. For example, higher flexibility and better work-life balance may explain higher reported levels of satisfaction with earnings in more digitalised occupations. Although company size might correspond with greater financial capacities to provide or improve aspects of objective job quality, several aspects of subjective job quality are found to be lower for employees working in larger companies. This may be due to higher anonymity and more rigid structures in larger companies. Such less attention may be paid to individual preferences and circumstances. Employees with high education and good assessed health have been found to report better job quality. We assume that those beneficial aspects translate into more advantageous working conditions and therefore in higher job satisfaction and less stress.

### Job Insecurity

Taking into account that some employees might fear losing their jobs due to substitution processes induced by digitalisation in the job insecurity model, the findings remain broadly as in the compositional model. Digitalisations associations with different aspects of subjective job quality do not change meaningfully. Nonetheless, subjective job insecurity proved an important control variable, as it was associated with lower levels of (aspects of) job satisfaction. The higher an employee’s job insecurity, the more stressed they feel about environmental factors and new responsibilities, and the more stressed they feel in general.

### Limitations

Since these analyses are based on cross-sectional data, it is not clear if digitalisation is associated with job satisfaction and perceived stress in the long term or if the associations discovered here are transitional. Future analysis using longitudinal data could shed light on this issue. Also, the concept of digitalisation of occupations based on substitution potential only indicates the potential degree of automation within occupations, not the extent to which occupations actually were automated. Hence, the concept relates more to technical feasibility than to the implementation of new technologies. Furthermore, substitution potential does not indicate an exact degree of digitalisation of single jobs. The realisation of the potential is very specific to the economic situation, organisational preferences or the management style of companies. The substitution potential data used in these analyses only approximate digitalisation in occupations. An additional limitation is the overrepresentation of high educated employees in the sample. As mentioned above, highly educated employees differ from lower educated regarding the occupation they are working in. However, the imbalance of the data regarding education bias results only in the pure model. In the other models, it is controlled for educational level.

## Conclusion

This work provides three key findings. First, for employees aged 40 and above in Germany, higher degrees of digitalisation of occupations correspond with predominantly higher subjective job quality. Second, the use of global measures or index measures to depict job quality can be misleading, since different aspects of job quality may show opposing trends which could cancel each other out at the aggregate level. Therefore, regarding subjective job quality and digitalisation, it is crucial to pay close attention to the multidimensionality of job quality. In order to develop effective measures to improve job quality, detailed and comprehensive insights are crucial. Third, the positive effects of digitalisation on job quality may not show up if compositional effects underlying the workforce are not controlled for, since employees working in occupations showing higher degrees of digitalisation may assess their job quality lower per se.

The adaption of new technologies puts new demands on employees and changes their everyday working life. The successful implementation of such technologies at work will, for sure, not take off by itself. Employees need to have the right skills to cope with emerging new work demands. The mismatch between employees’ skills and requirements at work is known as one important determinant for occupational switching (Guvenen et al., 2020) and job dissatisfaction (Shevchuk et al., 2019). In 2015 every fifth person aged 25–64 in Germany showed only little digital competencies (Eurostat, 2015). And close to every fourth person states that new technologies have been introduced to the workplace, but no further training have been provided (Initiative D21, 2021). We found lower satisfaction with training provision in more digitalised occupations in the pure model, but the association was not significant under control of compositional effects. Nonetheless and particularly for that reason further research on digitalisation and training participation is needed to reveal potential training gaps among different groups.

Recently, the COVID-19 pandemic has accelerated the digitalisation in the world of work. For many employees options like telework, online communication or e-commerce have enabled the continuation of work under statutory pandemic restrictions. Though, the sudden introduction of digital technology usage at work may have overwhelmed some employees. Potentials for improvements in the subjective job quality due to new technologies may have not been realised under these circumstances. Our results indicate that new technologies can improve the subjective job quality. However, the ongoing digitalisation in the world of work needs to be accompanied by strong employment regulations and provision of further training for all employees.

### Electronic supplementary material

Below is the link to the electronic supplementary material.

Supplementary file1 (PDF 426 KB)

## Data Availability

Data from the German Ageing Survey is freely available for the scientific community from the Research Data Centre of the German Centre of Gerontology (FDZ-DZA) upon request. Data on substitutability potentials are used with kind permission from the Institute of Employment Research (IAB).

## Appendix

See Table 1.

**Table 1:** Digitalisation and job quality, models 1–3. *Source* DEAS 2014. N = 1541

Job satisfaction		Index		…with work as a whole		…with earnings		…with the kind of work		…with working hours		…with career development or promotion		…with further training		…with the working atmosphere	
		b	SE	b	SE	b	SE	b	SE	b	SE	b	SE	b	SE	b	SE
Pure model	Digitalisation	0.009	0.068	0.092	0.077	- 0.034	0.103	- 0.004	0.085	**0.429**	0.099	–	–	** - 0.362**	0.127	- 0.020	0.093
Composition model	Digitalisation	**0.172**	0.073	**0.181**	0.085	**0.253**	0.109	0.069	0.094	**0.465**	0.104	**0.249**	0.124	- 0.109	0.144	0.103	0.104
Job insecurity model	Digitalisation	**0.188**	0.072	**0.198**	0.084	**0.266**	0.108	0.081	0.093	**0.473**	0.104	**0.274**	0.122	- 0.081	0.142	0.116	0.103
	Subj. job insecurity	** - 0.165**	0.024	** - 0.180**	0.028	** - 0.130**	0.036	** - 0.124**	0.031	** - 0.073**	0.035	** - 0.248**	0.040	** - 0.285**	0.048	** - 0.128**	0.034

Occupational stress		Index		…due to physical activities		…due to environmental factors		…due to tight schedules		…due to new responsibilities	
		b	SE	b	SE	b	SE	b	SE	b	SE
Pure model	Digitalisation	0.106	0.080	**0.381**	0.122	**0.810**	0.126	** - 0.618**	0.107	–	–
Composition model	Digitalisation	- 0.034	0.086	- 0.055	0.128	**0.345**	0.136	** - 0.518**	0.115	0.093	0.127
Job insecurity model	Digitalisation	- 0.040	0.086	- 0.060	0.128	**0.336**	0.136	** - 0.522**	0.115	0.085	0.127
	Subj. job insecurity	**0.065**	0.028	0.051	0.042	**0.091**	0.045	0.036	0.038	0.081	0.042

All models include the control variables gender, residency, age. From the composition model onwards it is controlled for: education, sector, company size, SRH, wage, working hours. The job insecurity model additionally controls for subjective job insecurity (1 = low–5 = high). Significant estimates (*p* < 0.05) in bold; estimates with *p* < 0.1 in italics; “−”: model is not significant. Rounded to three decimal places. Coefficients are unstandardised partial regression slopes

## Footnotes

1 The automation potentials determined by Arntz et al. (2016) for OECD countries are similar to substitution potentials determined by Dengler and Matthes (2018) for Germany.

2 Former Federal Republic of Germany excluding Berlin.

3 The highest correlation was found between working hours and gender (0.46), which can be assessed as “middle” correlation (Cohen, 1988).

4 Find a detailed presentation of the results in Tables 4–9 in the supplemental material.

## Code Availability

Code written in Stata 15; available upon reasonable request from the corresponding author.
