# Supplementary material for: Digitalisation and Employees’ Subjective Job Quality in the Second Half of Working Life in Germany
Source: Soc Indic Res. 2021 Dec 1;162(2):577–97. doi: 10.1007/s11205-021-02854-w (PMC8635314; doi:10.1007/s11205-021-02854-w)
Supplement: Supplementary file 1 — Supplementary file1 (PDF 426 KB) [file 11205_2021_2854_MOESM1_ESM.pdf]

Digitalisation and Employees' Subjective Job Quality in the Second Half of Working Life in Germany

Supplemental Material

1. Sample Description **2**

Tab. 1: Distribution of Selected Characteristics of the DEAS baseline sample 2014. 2

Tab. 2: Characteristics of Employees in Most/Least Digitalised Occupations. 2

Tab. 3: Comparison of Distributions among Different Levels of Self-Rated Health. 3

2. Analyses **4**

a) Pure Model

Tab. 4: Digitalisation and Job Satisfaction. 4

Tab. 5: Digitalisation and Occupational Stress. 4

b) Composition Model

Tab. 6: Digitalisation and Job Satisfaction Controlled for Compositional Effects. 5

Tab. 7: Digitalisation and Occupational Stress Controlled for Compositional Effects. 6

c) Job Insecurity Model

Tab. 8: Digitalisation and Job Satisfaction Controlled for Job Insecurity. 7

Tab. 9: Digitalisation and Occupational Stress Controlled for Job Insecurity. 8

3. Further Analyses **9**

Tab. 10: Digitalisation and Job Satisfaction with Clustered Standard Errors for Occupations. 9

Tab. 11: Digitalisation and Occupational Stress with Clustered Standard Errors for Occupations 10

Tab. 12: Digitalisation and Job Satisfaction without Controlling for Self-Rated Health. 11

Tab. 13: Digitalisation and Occupational Stress without Controlling for Self-Rated Health 12

4. Hypotheses **13**

Tab. 14: Overview of all Formulated Hypotheses and Related Results. 13

1. Sample Description

Table 1: Distribution of Selected Characteristics of the DEAS Baseline Sample 2014.

**gender** %		**company size** %	
female	49	less than 5	6
male	51	5 to 20 employees	19
**education (ISCED)** %		21 to 100 employees	24
low	3	101 to 200 employees	12
middle	53	201 to 2000 employees	23
high	44	2000 and more	16
**region** %		**sector** %	
East Germany	32	agriculture & forestry	1
West Germany	68	industry	22
**age** Ø		handicraft	9
Mean	52	commercial or service	44
SD	6.15	public service	24

Source: DEAS 2014; employees subject to social insurance contributions; N = 1541.

Table 2: Characteristics of Employees in Most/Least Digitalised Occupations.

**most digitalised occupations (>=70%)**				**least digitalised occupations (<30%)**			
**gender** %		**company size** %		**gender** %		**company size** %	
female	30	less than 5	6	female	50	less than 5	6
male	70	5 to 20 employees	12	male	49	5 to 20 employees	12
**education (ISCED)** %		21 to 100 employees	22	**education (ISCED)** %		21 to 100 employees	22
low	5	101 to 200 employees	11	low	4	101 to 200 employees	11
middle	78	201 to 2000 employees	31	middle	42	201 to 2000 employees	31
high	17	2000 and more	18	high	54	2000 and more	18
**region** %		**sector** %		**region** %		**sector** %	
East Germany	28	agriculture & forestry	1	East Germany	32	agriculture & forestry	1
West Germany	72	industry	53	West Germany	68	industry	15
		handicraft	17			handicraft	6
**age** Ø	52	commercial or service	21	**age** Ø	52	commercial or service	46
**wage (€/h)** Ø	11	public service	8	**wage (€/h)** Ø	13	public service	32
**working hours (week)** Ø	39			**working hours (week)** Ø	40		

Source: DEAS 2014; employees subject to social insurance contributions. Most digital occupations (>=70%): N = 174; least digitalised occupations (<30%): N = 733. Numbers rounded to integers.

Table 3: Comparison of Distributions among Different Levels of Self-Rated Health.

**self-rated health**	Our sample (n = 1541)	DEAS baseline sample; 40–65 years (n = 1850)
very bad	0.59%	0.70%
bad	5.66%	5.68%
middle	29.76%	29.94%
good	51.67%	52.00%
very good	12.32%	11.68%

Source: DEAS 2014.

2. Analyses

a) Pure Model

Table 4: Digitalisation and Job Satisfaction.

job satisfaction	(1) index		(2) … with work as a whole		(3) … with earnings		(4) … with the kind of work		(5) … with working hours		(6) … with opp. for career development or promotion		(7) … with opp. for further training		(8) … with working atmosphere	
	b	SE	b	SE	b	SE	b	SE	b	SE	b	SE	b	SE	b	SE
digitalisation	0.009	0.068	0.092	0.077	-0.034	0.103	-0.004	0.085	**0.429**	0.099	0.049	0.111	**-0.362**	0.127	-0.020	0.093
male	-0.030	0.035	-0.047	0.041	**0.123**	0.053	0.011	0.044	**-0.182**	0.052	-0.047	0.058	-0.101	0.066	0.016	0.049
age (in years)	<0.001	0.003	0.004	0.003	<0.001	0.004	0.001	0.004	*0.007*	0.004	0.003	0.005	0.006	0.005	**-0.013**	0.004
West Germany	**0.165**	0.038	**0.180**	0.043	**0.285**	0.057	**0.147**	0.047	**0.245**	0.055	0.063	0.061	**0.142**	0.070	**0.110**	0.052
constant	**3.710**	0.154	**3.840**	0.177	**3.384**	0.232	**4.073**	0.192	**3.378**	0.225	**3.407**	0.251	**3.306**	0.283	**4.712**	0.212
observations	1541		1541		1541		1541		1541		1541		1541		1541	
prob > F	0.001		0.000		0.000		0.047		0.000		0.701		0.003		0.004	

Source: DEAS 2014. Notes: Coefficients are unstandardised partial regression slopes. Significant estimates (p<0.05) in bold; estimates with p<0.1 in italics. Rounded to three decimal places.

Table 5: Digitalisation and Occupational Stress.

occupational stress	(1) index		(2) … due to physical activities		(3) … due to negative environmental factors		(4) … due to tight schedules		(5) … due to new responsibilities	
	b	SE	b	SE	b	SE	b	SE	b	SE
digitalisation	0.106	0.080	**0.381**	0.122	**0.810**	0.126	**-0.618**	0.107	-0.152	0.116
male	0.051	0.041	0.007	0.063	*0.108*	0.065	0.074	0.056	0.015	0.060
age (in years)	0.001	0.003	*0.010*	0.005	-0.007	0.005	<0.001	0.005	0.002	0.005
West Germany	**-0.300**	0.044	**-0.523**	0.068	**-0.314**	0.070	**-0.278**	0.059	-0.087	0.065
constant	**2.930**	0.180	**2.614**	0.276	**2.555**	0.285	**3.817**	0.243	**2.733**	0.263
observations	1541		1541		1541		1541		1541	
prob > F	0.000		0.000		0.000		0.000		0.421	

Source: DEAS 2014. Notes: Coefficients are unstandardised partial regression slopes. Significant estimates (p<0.05) in bold; estimates with p<0.1 in italics. Rounded to three decimal places.

b) Composition Model

Table 6: Digitalisation and Job Satisfaction Controlled for Compositional Effects.

	(1)		(2)		(3)		(4)		(5)		(6)		(7)		(8)	
job satisfaction	index		… with work as a whole		… with earnings		… with the kind of work		… with working hours		… with opp. for career development or promotion		… with opp. for further training		… with working atmosphere	
	b	SE	b	SE	b	SE	b	SE	b	SE	b	SE	b	SE	b	SE
digitalisation	**0.172**	0.073	**0.181**	0.085	**0.253**	0.109	0.069	0.094	**0.465**	0.104	**0.249**	0.124	-0.109	0.144	0.103	0.104
male	*-0.072*	0.041	*-0.083*	0.048	-0.062	0.061	-0.077	0.052	0.058	0.059	**-0.194**	0.068	**-0.169**	0.078	0.013	0.058
age (in years)	0.001	0.003	0.005	0.003	-0.001	0.004	0.002	0.004	**0.008**	0.004	0.002	0.005	0.005	0.005	**-0.011**	0.004
West Germany	**0.085**	0.038	**0.149**	0.045	**0.115**	0.056	**0.106**	0.049	**0.116**	0.054	0.028	0.064	0.054	0.071	*0.091*	0.054
education (ref.: low)																
middle	0.022	0.096	-0.067	0.113	-0.071	0.143	-0.045	0.123	-0.057	0.138	0.208	0.160	0.145	0.184	-0.048	0.137
high	0.023	0.098	-0.092	0.116	-0.079	0.146	-0.067	0.126	0.042	0.141	0.106	0.165	0.107	0.188	0.033	0.141
sector (ref.: agriculture & forestry)																
industry	0.076	0.158	0.128	0.187	-0.088	0.235	0.064	0.203	0.092	0.228	0.149	0.265	0.048	0.311	0.189	0.225
handicraft	0.180	0.162	0.228	0.191	-0.082	0.240	0.110	0.207	0.241	0.233	0.308	0.271	0.082	0.320	*0.422*	0.229
commercial & service	0.078	0.155	0.171	0.183	-0.144	0.230	0.018	0.198	-0.005	0.223	0.194	0.258	0.121	0.306	0.281	0.219
public service	0.193	0.158	0.200	0.186	0.013	0.233	0.051	0.202	0.157	0.227	0.362	0.262	0.309	0.312	0.268	0.224
company size	**-0.032**	0.013	**-0.029**	0.015	0.018	0.019	-0.020	0.016	**-0.068**	0.018	**-0.042**	0.021	-0.013	0.025	**-0.068**	0.018
self-rated health	**0.154**	0.022	**0.184**	0.026	**0.159**	0.033	**0.196**	0.028	**0.219**	0.032	0.057	0.037	**0.112**	0.042	**0.183**	0.031
hourly wage (€/h)	**0.032**	0.004	**0.016**	0.005	**0.060**	0.006	**0.019**	0.005	**0.025**	0.006	**0.035**	0.007	**0.038**	0.007	**0.016**	0.006
working hours (per week)	-0.001	0.002	0.003	0.002	0.003	0.003	*0.004*	0.002	**-0.029**	0.003	**0.011**	0.003	0.003	0.004	0.001	0.003
constant	**2.787**	0.261	**2.799**	0.306	**2.192**	0.386	**2.994**	0.332	**3.501**	0.373	**2.187**	0.437	**2.147**	0.504	**3.701**	0.368
observations	1541		1541		1541		1541		1541		1541		1541		1541	
prob > F	0.000		0.000		0.000		0.000		0.000		0.000		0.000		0.000	

Source: DEAS 2014. Notes: Coefficients are unstandardised partial regression slopes. Significant estimates (p<0.05) in bold; estimates with p<0.1 in italics. Rounded to three decimal places. Self-rated health: 1=low–5=high.

Table 7: Digitalisation and Occupational Stress Controlled for Compositional Effects.

occupational stress	(1) index		(2) … due to physical activities		(3) … due to negative environmental factors		(4) … due to tight schedules		(5) … due to new responsibilities	
	b	SE	b	SE	b	SE	b	SE	b	SE
digitalisation	-0.034	0.086	-0.055	0.128	**0.345**	0.136	**-0.518**	0.115	0.093	0.127
male	-0.056	0.047	0.058	0.070	0.104	0.075	**-0.194**	0.064	**-0.191**	0.070
age (in years)	-0.002	0.003	0.008	0.005	**-0.010**	0.005	-0.004	0.004	-0.002	0.005
West Germany	**-0.236**	0.044	**-0.369**	0.066	**-0.251**	0.070	**-0.211**	0.059	*-0.112*	0.066
education (ref.: low)										
middle	-0.044	0.110	*-0.308*	0.166	**-0.363**	0.175	0.195	0.150	*0.298*	0.165
high	-0.142	0.113	**-0.682**	0.170	**-0.655**	0.179	**0.316**	0.154	**0.452**	0.169
sector (ref.: agriculture & forestry)										
industry	-0.073	0.183	-0.126	0.275	-0.389	0.289	0.171	0.246	0.052	0.272
handicraft	-0.092	0.187	0.112	0.280	-0.134	0.295	-0.151	0.252	-0.197	0.278
commercial & service	-0.217	0.179	-0.184	0.269	**-0.777**	0.282	0.056	0.240	0.037	0.265
public service	-0.142	0.182	-0.184	0.273	**-0.592**	0.287	0.081	0.245	0.125	0.270
company size	**0.062**	0.015	0.016	0.022	**0.077**	0.023	**0.073**	0.020	**0.083**	0.022
self-rated health	**-0.287**	0.025	**-0.342**	0.038	**-0.273**	0.040	**-0.307**	0.035	**-0.226**	0.038
hourly wage (€/h)	**-0.020**	0.005	**-0.049**	0.007	**-0.036**	0.007	-0.009	0.006	**0.015**	0.007
working hours (per week)	**0.009**	0.002	0.005	0.003	-0.005	0.004	**0.024**	0.003	**0.013**	0.003
constant	**4.107**	0.300	**4.953**	0.451	**5.238**	0.476	**3.833**	0.404	**2.404**	0.447
observations	1541		1541		1541		1541		1541	
prob > F	0.000		0.000		0.000		0.000		0.000	

Source: DEAS 2014. Notes: Coefficients are unstandardised partial regression slopes. Significant estimates (p<0.05) in bold; estimates with p<0.1 in italics. Rounded to three decimal places. Self-rated health: 1=low–5=high.

c) Job Insecurity Model

Table 8: Digitalisation and Job Satisfaction Controlled for Job Insecurity.

job satisfaction	(1) index		(2) … with work as a whole		(3) … with earnings		(4) … with the kind of work		(5) … with working hours		(6) … with opp. for career development or promotion		(7) … with opp. for further training		(8) … with working atmosphere	
	b	SE	b	SE	b	SE	b	SE	b	SE	b	SE	b	SE	b	SE
digitalisation	**0.188**	0.072	**0.198**	0.084	**0.266**	0.108	0.081	0.093	**0.473**	0.104	**0.274**	0.122	-0.081	0.142	0.116	0.103
subj. job insecurity	**-0.165**	0.024	**-0.180**	0.028	**-0.130**	0.036	**-0.124**	0.031	**-0.073**	0.035	**-0.248**	0.040	**-0.285**	0.048	**-0.128**	0.034
male	-0.063	0.040	-0.074	0.047	-0.055	0.061	-0.071	0.052	0.061	0.059	**-0.181**	0.067	**-0154**	0.078	0.019	0.057
age (in years)	<0.001	0.003	0.004	0.003	-0.002	0.004	0.001	0.004	*0.008*	0.004	<0.001	0.005	0.003	0.005	**-0.012**	0.004
West Germany	*0.065*	0.037	**0.127**	0.044	*0.099*	0.056	*0.090*	0.048	*0.107*	0.055	-0.003	0.063	0.019	0.071	0.076	0.054
education (ref.: low)																
middle	0.045	0.094	-0,042	0.112	-0.053	0.142	-0.028	0.123	-0.047	0.138	0.242	0.158	0.184	0.182	-0.030	0.137
high	0.052	0.097	-0.061	0.115	-0.057	0.146	-0.045	0.126	0.055	0.141	0.149	0.163	0.156	0.185	0.055	0.140
sector (ref.: agriculture & forestry)																
industry	0.131	0.156	0.187	0.185	-0.044	0.234	0.105	0.202	0.116	0.228	0.232	0.262	0.143	0.308	0.232	0.225
handicraft	0.201	0.160	0.251	0.189	-0.066	0.239	0.126	0.206	0.250	0.232	0.340	0.269	0.119	0.317	*0.438*	0.229
commercial & service	0.115	0.153	0.211	0.180	-0.115	0.229	0.046	0.197	0.012	0.223	0.250	0.255	0.185	0.303	0.310	0.219
public service	0.189	0.155	0.196	0.184	0.010	0.232	0.048	0.201	0.155	0.226	0.356	0.259	0.301	0.309	0.265	0.223
company size	**-0.036**	0.013	**-0.033**	0.015	0.016	0.019	-0.022	0.016	**-0.070**	0.018	**-0.047**	0.021	-0.019	0.024	**-0.071**	0.018
self-rated health	**0.143**	0.022	**0.171**	0.026	**0.150**	0.033	**0.187**	0.028	**0.214**	0.032	0.039	0.037	**0.092**	0.042	**0.174**	0.031
hourly wage (€/h)	**0.029**	0.004	**0.012**	0.005	**0.058**	0.006	**0.017**	0.005	**0.023**	0.006	**0.030**	0.007	**0.032**	0.007	**0.014**	0.006
working hours (per week)	-0.001	0.002	0.003	0.002	0.003	0.003	*0.004*	0.002	**-0.029**	0.003	**0.011**	0.003	0.002	0.004	<0.001	0.003
constant	**3.174**	0.262	**3.221**	0.309	**2.496**	0.393	**3.286**	0.338	**3.673**	0.381	**2.769**	0.444	**2.817**	0.508	**4.002**	0.374
observations	1541		1541		1541		1541		1541		1541		1541		1541	
prob > F	0.000		0.000		0.000		0.000		0.000		0.000		0.000		0.000	

Source: DEAS 2014. Notes: Coefficients are unstandardised partial regression slopes. Significant estimates (p<0.05) in bold; estimates with p<0.1 in italics. Rounded to three decimal places. Self-rated health and job insecurity: 1=low–5=high.

Table 9: Digitalisation and Occupational Stress Controlled for Job Insecurity.

occupational stress	(1) index		(2) … due to physical activities		(3) … due to negative environmental factors		(4) … due to tight schedules		(5) … due to new responsibilities	
	b	SE	b	SE	b	SE	b	SE	b	SE
digitalisation	-0.040	0.086	-0.060	0.128	**0.336**	0.136	**-0.522**	0.115	0.085	0.127
subj. job insecurity	**0.065**	0.028	0.051	0.042	**0.091**	0.045	0.036	0.038	*0.081*	0.042
male	-0.059	0.047	0.055	0.070	0.100	0.075	**-0.196**	0.064	**-0.195**	0.070
age (in years)	-0.002	0.003	*0.008*	0.005	*-0.009*	0.005	-0.004	0.004	-0.001	0.050
West Germany	**-0.288**	0.044	**-0.363**	0.066	**-0.240**	0.070	**-0.207**	0.059	-0.101	0.066
education (ref.: low)										
middle	-0.053	0.110	*-0.315*	0.166	**-0.375**	0.175	0.190	0.150	*0.287*	0.165
high	-0.154	0.113	**-0.069**	0.170	**-0.671**	0.179	**0.310**	0.154	**0.438**	0.169
sector (ref.: agriculture & forestry)										
industry	-0.095	0.183	-0.143	0.275	-0.420	0.289	0.159	0.247	0.025	0.272
handicraft	-0.101	0.186	0.105	0.280	-0.145	0.294	-0.155	0.252	-0.207	0.278
commercial & service	-0.232	0.179	-0.196	0.269	**-0.797**	0.282	0.048	0.241	0.019	0.265
public service	-0.141	0.182	-0.183	0.273	**-0.590**	0.287	0.082	0.245	0.128	0.270
company size	**0.064**	0.015	0.017	0.040	**0.079**	0.023	**0.074**	0.020	**0.085**	0.022
self-rated health	**-0.283**	0.025	**-0.339**	0.038	**-0.267**	0.040	**-0.305**	0.035	**-0.220**	0.038
hourly wage (€/h)	**-0.019**	0.005	**-0.048**	0.007	**-0.034**	0.007	-0.009	0.006	**0.016**	0.007
working hours (per week)	**0.009**	0.002	0.005	0.003	-0.005	0.004	**0.024**	0.003	**0.014**	0.003
constant	**3.955**	0.306	**4.833**	0.460	**5.025**	0.487	**3.749**	0.414	**2.213**	0.457
observations	1541		1541		1541		1541		1541	
prob > F	0.000		0.000		0.000		0.000		0.000	

Source: DEAS 2014. Notes: Coefficients are unstandardised partial regression slopes. Significant estimates (p<0.05) in bold; estimates with p<0.1 in italics. Rounded to three decimal places. Self-rated health and job insecurity: 1=low–5=high.

3. Additional Analyses

In order to check the robustness of our analyses further estimations based on model 3 (job insecurity model) have been made. We re-estimated our model (1) using clustered standard errors for occupation and (2) without controlling for self-rated health. The findings of the re-estimation with clustered standard errors is very similar to the findings of the original job insecurity model. This indicates a good quality of the imputation of missing values on the digitalisation variable. Likewise the findings of the re-estimation without the consideration of the control variable self-rated health are similar to the findings the original model. Self-rated health does not seem to vary greatly between employees in more/less digitalised occupations.

Table 10: Digitalisation and Job Satisfaction with Clustered Standard Errors for Occupation.

job satisfaction	(1) index		(2) … with work as a whole		(3) … with earnings		(4) … with the kind of work		(5) … with working hours		(6) … with opp. for career development or promotion		(7) … with opp. for further training		(8) … with working atmosphere	
	b	SE	b	SE	b	SE	b	SE	b	SE	b	SE	b	SE	b	SE
digitalisation	*0.188*	0.096	*0.198*	0.102	**0.266**	0.124	0.081	0.120	**0.473**	0.100	**0.274**	0.138	-0.081	0.182	0.116	0.131
subj. job insecurity	**-0.165**	0.024	**-0.180**	0.034	**-0.130**	0.041	**-0.124**	0.033	*-0.073*	0.038	**-0.248**	0.042	**-0.285**	0.047	**-0.128**	0.037
male	-0.063	0.043	-0.074	0.048	-0.055	0.071	-0.071	0.057	0.061	0.062	**-0.181**	0.067	*-0.154*	0.082	0.019	0.051
age (in years)	<0.001	0.003	0.004	0.003	-0.002	0.004	0.001	0.004	**0.008**	0.004	<0.001	0.005	0.003	0.005	**-0.012**	0.004
West Germany	0.065	0.044	**0.127**	0.048	0.099	0.062	0.090	0.057	*0.107*	0.059	-0.003	0.066	0.019	0.075	0.076	0.049
education (ref.: low)																
middle	0.045	0.079	-0.042	0.116	-0.053	0.192	-0.028	0.123	-0.047	0.120	*0.242*	0.146	0.184	0.170	-0.030	0.114
high	0.052	0.085	-0.061	0.123	-0.057	0.199	-0.045	0.124	0.055	0.125	0.149	0.154	0.156	0.180	0.055	0.112
sector (ref.: agriculture & forestry)																
industry	0.131	0.178	0.187	0.194	-0.044	0.281	0.105	0.146	0.116	0.254	0.232	0.303	0.143	0.281	0.232	0.269
handicraft	0.201	0.176	0.251	0.186	-0.066	0.277	0.126	0.144	0.250	0.249	0.340	0.305	0.119	0.300	*0.438*	0.255
commercial & service	0.115	0.173	0.211	0.184	-0.115	0.270	0.046	0.141	0.012	0.252	0.250	0.297	0.185	0.281	0.310	0.257
public service	0.189	0.172	0.196	0.194	0.010	0.270	0.048	0.144	0.155	0.251	0.356	0.296	0.301	0.284	0.265	0.262
company size	**-0.036**	0.013	**-0.033**	0.014	0.016	0.019	-0.022	0.017	**-0.070**	0.016	**-0.047**	0.021	-0.019	0.027	**-0.071**	0.020
self-rated health	**0.143**	0.023	**0.171**	0.029	**0.150**	0.032	**0.187**	0.034	**0.214**	0.033	0.039	0.037	**0.092**	0.042	**0.174**	0.037
hourly wage (€/h)	**0.029**	0.005	**0.012**	0.005	**0.058**	0.008	**0.017**	0.005	**0.023**	0.006	**0.030**	0.007	**0.032**	0.007	**0.014**	0.006
working hours (per week)	-0.001	0.002	0.003	0.002	0.003	0.003	0.004	0.003	**-0.029**	0.003	**0.011**	0.003	0.002	0.004	<0.001	0.003
constant	**3.174**	0.293	**3.221**	0.315	**2.496**	0.447	**3.286**	0.370	**3.673**	0.437	**2.769**	0.455	**2.817**	0.552	**4.002**	0.374
observations	1541		1541		1541		1541		1541		1541		1541		1541	
prob > F	0.000		0.000		0.000		0.000		0.000		0.000		0.000		0.000	

Source: DEAS 2014. Notes: Estimations based on the job insecurity model. Coefficients are unstandardised partial regression slopes. Significant estimates (p<0.05) in bold; estimates with p<0.1 in italics. Rounded to three decimal places. Self-rated health and job insecurity: 1=low–5=high.

Table 11: Digitalisation and Occupational Stress with Clustered Standard Errors for Occupation.

occupational stress	(1) index		(2) … due to physical activities		(3) … due to negative environmental factors		(4) … due to tight schedules		(5) … due to new responsibilities	
	b	SE	b	SE	b	SE	b	SE	b	SE
digitalisation	-0.040	0.125	-0.060	0.204	0.336	0.209	**-0.522**	0.135	0.085	0.139
subj. job insecurity	**0.065**	0.027	0.051	0.042	*0.091*	0.048	0.036	0.035	**0.081**	0.039
male	-0.059	0.063	0.055	0.096	0.100	0.093	**-0.196**	0.082	**-0.195**	0.078
age (in years)	-0.002	0.003	0.008	0.005	**-0.009**	0.005	-0.004	0.005	-0.001	0.005
West Germany	**-0.228**	0.049	**-0.363**	0.062	**-0.240**	0.078	**-0.207**	0.058	-0.101	0.076
education (ref.: low)										
middle	-0.053	0.112	*-0.315*	0.166	**-0.375**	0.161	0.190	0.181	*0.287*	0.168
high	-0.154	0.111	**-0.690**	0.164	**-0.671**	0.177	*0.310*	0.181	0.438	0.167
sector (ref.: agriculture & forestry)										
industry	-0.095	0.152	-0.143	0.293	-0.420	0.298	0.159	0.224	0.025	0.250
handicraft	-0.101	0.157	0.105	0.307	-0.145	0.303	-0.155	0.231	-0.207	0.256
commercial & service	-0.232	0.149	-0.196	0.301	**-0.797**	0.290	0.048	0.219	0.019	0.243
public service	-0.141	0.156	-0.182	0.310	*-0.590*	0.301	0.082	0.229	0.128	0.243
company size	**0.064**	0.019	0.017	0.024	**0.079**	0.031	**0.074**	0.024	**0.085**	0.025
self-rated health	**-0.283**	0.024	**-0.339**	0.037	**-0.267**	0.041	**-0.305**	0.033	**-0.220**	0.043
hourly wage (€/h)	**-0.019**	0.004	**-0.048**	0.007	**-0.034**	0.009	-0.009	0.006	**0.016**	0.008
working hours (per week)	**0.009**	0.003	0.005	0.004	-0.005	0.004	**0.024**	0.003	**0.014**	0.003
constant	**3.955**	0.281	**4.833**	0.472	**5.025**	0.455	**3.749**	0.418	**2.213**	0.470
observations	1541		1541		1541		1541		1541	
prob > F	0.000		0.000		0.000		0.000		0.000	

Source: DEAS 2014. Notes: Estimations based on the job insecurity model. Coefficients are unstandardised partial regression slopes. Significant estimates (p<0.05) in bold; estimates with p<0.1 in italics. Rounded to three decimal places. Self-rated health and job insecurity: 1=low–5=high.

Table 12: Digitalisation and Job Satisfaction without Controlling for Self-Rated Health.

	(1)		(2)		(3)		(4)		(5)		(6)		(7)		(8)	
job satisfaction	index		… with work as a whole		… with earnings		… with the kind of work		… with working hours		… with opp. for career development or promotion		… with opp. for further training		… with working atmosphere	
	b	SE	b	SE	b	SE	b	SE	b	SE	b	SE	b	SE	b	SE
digitalisation	**0.200**	0.073	**0.213**	0.085	**0.279**	0.109	0.098	0.095	**0.491**	0.105	**0.277**	0.122	-0.073	0.142	0.131	0.104
subj. job insecurity	**-0.177**	0.024	**-0.194**	0.029	**-0.142**	0.036	**-0.140**	0.031	**-0.091**	0.035	**-0.251**	0.040	**-0.293**	0.047	**-0.143**	0.035
male	*-0.075*	0.040	*-0.087*	0.048	-0.067	0.061	-0.085	0.053	0.045	0.059	**-0.184**	0.067	**-0.161**	0.078	0.006	0.058
age (in years)	-0.002	0.003	0.002	0.003	-0.004	0.004	-0.001	0.004	0.005	0.004	-0.001	0.005	0.002	0.005	**-0.014**	0.004
West Germany	*0.065*	0.038	**0.126**	0.045	*0.099*	0.057	*0.090*	0.049	*0.107*	0.055	-0.003	0.063	0.019	0.071	0.075	0.054
education (ref.: low)																
middle	0.049	0.096	-0.037	0.113	-0.049	0.144	-0.022	0.124	-0.041	0.140	0.243	0.158	0.187	0.182	-0.026	0.138
high	0.075	0.098	-0.034	0.116	-0.033	0.147	-0.016	0.128	0.089	0.143	0.155	0.163	0.171	0.189	0.082	0.141
sector (ref.: agriculture & forestry)																
industry	0.175	0.159	0.241	0.188	0.002	0.235	0.163	0.205	0.182	0.232	0.244	0.262	0.172	0.308	0.286	0.227
handicraft	0.245	0.162	0.304	0.191	-0.019	0.240	0.184	0.209	0.316	0.236	0.352	0.269	0.147	0.317	**0.492**	0.231
commercial & service	0.158	0.155	0.263	0.183	-0.069	0.230	0.103	0.200	0.077	0.226	0.261	0.255	0.213	0.303	0.363	0.221
public service	0.229	0.157	0.244	0.186	0.051	0.234	0.101	0.204	0.215	0.230	0.367	0.259	0.327	0.308	0.313	0.225
company size	**-0.036**	0.013	**-0.033**	0.015	0.015	0.019	-0.023	0.016	**-0.070**	0.018	**-0.047**	0.021	-0.019	0.024	**-0.071**	0.018
hourly wage (€/h)	0.031	0.004	0.014	0.005	0.060	0.006	0.019	0.005	0.026	0.006	0.031	0.007	0.033	0.007	0.016	0.006
working hours (per week)	-0.001	0.002	0.003	0.002	0.003	0.003	*0.005*	0.002	**-0.029**	0.003	**0.011**	0.003	0.002	0.004	0.001	0.003
constant	3.735	0.252	3.894	0.375	3.086	0.375	4.022	0.324	4.513	0.366	3.179	0.420	3.179	0.484	4.686	0.358
observations	1541		1541		1541		1541		1541		1541		1541		1541	
prob > F	0.000		0.000		0.000		0.000		0.000		0.000		0.000		0.000	

Source: DEAS 2014. Notes: Coefficients are unstandardised partial regression slopes. Significant estimates (p<0.05) in bold; estimates with p<0.1 in italics. Rounded to three decimal places. Job insecurity: 1=low–5=high.

Table 13: Digitalisation and Occupational Stress without Controlling for Self-Rated Health.

occupational stress	(1) index		(2) … due to physical activities		(3) … due to negative environmental factors		(4) … due to tight schedules		(5) … due to new responsibilities	
	b	SE	b	SE	b	SE	b	SE	b	SE
digitalisation	-0.065	0.089	-0.089	0.131	**0.313**	0.138	**-0.548**	0.118	0.066	0.128
subj. job insecurity	**0.089**	0.029	*0.079*	0.043	**0.113**	0.045	0.061	0.039	**0.100**	0.043
male	-0.037	0.049	0.082	0.072	0.121	0.076	**-0.172**	0.065	**-0.178**	0.071
age (in years)	0.002	0.003	**0.013**	0.005	-0.006	0.005	<0.001	0.004	0.002	0.005
West Germany	**-0.288**	0.046	**-0.363**	0.068	**-0.240**	0.071	**-0.206**	0.061	-0.101	0.066
education (ref.: low)										
middle	-0.182	0.115	*-0.324*	0.171	-0.383	0.178	0.182	0.154	*0.281*	0.167
high	*-0.198*	0.118	**-0.744**	0.175	**-0.723**	0.182	*0.262*	0.158	**0.403**	0.171
sector (ref.: agriculture & forestry)										
industry	-0.182	0.190	-0.248	0.283	*-0.502*	0.293	0.064	0.253	-0.043	0.275
handicraft	-0.188	0.194	0.001	0.288	-0.228	0.298	-0.250	0.258	-0,275	0.280
commercial & service	*-0.317*	0.186	-0.299	0.276	**-0.878**	0.286	-0.045	0.247	-0.048	0.268
public service	-0.220	0.189	-0.277	0.281	**-0.664**	0.291	-0.003	0.251	0.066	0.273
company size	**0.064**	0.015	0.018	0.023	**0.079**	0.024	**0.075**	0.021	**0.085**	0.022
hourly wage (€/h)	**-0.022**	0.005	**-0.053**	0.007	**-0.037**	0.008	*-0.013*	0.007	*0.014*	0.007
working hours (per week)	**0.009**	0.002	0.005	0.003	-0.005	0.004	**0.023**	0.003	**0.013**	0.003
constant	**2.844**	0.301	**3.501**	0.446	**3.977**	0.466	**2.551**	0.401	**1.347**	0.437
observations	1541		1541		1541		1541		1541	
prob > F	0.000		0.000		0.000		0.000		0.000	

Source: DEAS 2014. Notes: Coefficients are unstandardised partial regression slopes. Significant estimates (p<0.05) in bold; estimates with p<0.1 in italics. Rounded to three decimal places. Job insecurity: 1=low–5=high.

4. Hypotheses

Table 14: Overview of all Formulated Hypotheses and Related Results.

Higher degrees of digitalisation in the employees occupation			
**1. Pure Model**		Hypothesis	Finding
H1.1	job satisfaction index	-	n.s.
H1.2	job satisfaction as a whole	-	n.s.
**H1.a**	job satisfaction with opportunities for further training	**-**	**-**
	job satisfaction with working hours	e	+
	job satisfaction with the working atmosphere	e	n.s.
	job satisfaction with the kind of work	e	n.s.
	job satisfaction with earnings	e	n.s.
	job satisfaction with career development and promotion	e	n.s.
H2	occupational stress index	+	n.s.
**H2.a**	occupational stress due to physical work	**+**	**+**
**H2.b**	occupational stress due to negative environmental factors	**+**	**+**
	occupational stress due to tight schedules	e	-
	occupational stress due to new responsibilities	e	n.s.

Higher degrees of digitalisation in the employees occupation			
**2. Compositional Model**		Hypothesis	Finding
**H3.1**	job satisfaction index	**+**	**+**
**H3.2**	job satisfaction as a whole	**+**	**+**
H3.a	job satisfaction with opportunities for further training	-	n.s.
**H3.b**	job satisfaction with working hours	**+**	**+**
H3.c	job satisfaction with the working atmosphere	+	n.s.
H3.d	job satisfaction with the kind of work	+	n.s.
	job satisfaction with earnings	e	+
	job satisfaction with career development and promotion	e	+
H4	occupational stress index	-	n.s.
H4.a	occupational stress due to physical work	-	n.s.
H4.b	occupational stress due to negative environmental factors	-	+
H4.c	occupational stress due to tight schedules	+	-
H4.d	occupational stress due to new responsibilities	+	n.s.

Higher degrees of digitalisation in the employees occupation			
**3. Job Insecurity Model**		Hypothesis	Finding
**H5.1**	job satisfaction index	**+**	**+**
**H5.2**	job satisfaction as a whole	**+**	**+**
H5.a	job satisfaction with opportunities for further training	-	n.s.
**H5.b**	job satisfaction with working hours	**+**	**+**
H5.c	job satisfaction with the working atmosphere	+	n.s.
H5.d	job satisfaction with the kind of work	+	n.s.
	job satisfaction with earnings	e	+
	job satisfaction with career development and promotion	e	+
H6	occupational stress index	-	n.s.
H6.a	occupational stress due to physical work	-	n.s.
H6.b	occupational stress due to negative environmental factors	-	+
H6.c	occupational stress due to tight schedules	+	-
H6.d	occupational stress due to new responsibilities	+	n.s.

Higher degrees of subjective job insecurity			
**3. Job Insecurity Model**		Hypothesis	Finding
**H7.1**	job satisfaction index	**-**	**-**
**H7.2**	job satisfaction as a whole	**-**	**-**
**H7.a**	job satisfaction with opportunities for further training	**-**	**-**
**H7.b**	job satisfaction with working hours	**-**	**-**
**H7.c**	job satisfaction with the working atmosphere	**-**	**-**
**H7.d**	job satisfaction with the kind of work	**-**	**-**
**H7.e**	job satisfaction with earnings	**-**	**-**
**H7.f**	job satisfaction with career development and promotion	**-**	**-**
**H8**	occupational stress index	**+**	**+**
H8.a	occupational stress due to physical work	+	n.s.
**H8.b**	occupational stress due to negative environmental factors	**+**	**+**
H8.c	occupational stress due to tight schedules	+	n.s.
**H8.d**	occupational stress due to new responsibilities	**+**	**(+)**

Note: e = exploratory approach; +/- positive/negative association (significant with $p<0.05$); (+)/(-) positive/negative association (significant with $p<0.1$); n.s. not significant. Bold letters indicate that the hypotheses was confirmed by our findings.
